# Community and stakeholders’ engagement in the prevention and management of Type 2 diabetes: a qualitative study in socioeconomically disadvantaged suburbs in region Stockholm

**DOI:** 10.1080/16549716.2019.1609313

**Published:** 2019-05-22

**Authors:** F. Al-Murani, J. Aweko, I. Nordin, P. Delobelle, Fx. Kasujja, C.-G. Östenson, S. S. Peterson, M. Daivadanam, HM. Alvesson

**Affiliations:** a Department of Public Health Sciences, Karolinska Institutet, Stockholm, Sweden; b Department of Food Studies, Nutrition, and Dietetics, Uppsala University, Uppsala, Sweden; c School of Public Health, University of the Western Cape, Bellville, South Africa; d Chronic Disease Initiative for Africa, University of Cape Town, Rondebosch, South Africa; e Department of Biostatistics and Epidemiology, Makerere University College of Health Sciences, School of Public Health, Kampala, Uganda; f Department of Molecular Medicine and Surgery, Diabetes and Endocrinology Unit, Karolinska Institutet, Stockholm, Sweden; g Department of Women‘s and Children‘s Health, International Maternal and Child Health, Uppsala University, Uppsala, Sweden

**Keywords:** Community, Type 2 diabetes, health promotion, NCD prevention, qualitative studies

## Abstract

**Background**: Community-based approaches have been identified as an effective strategy to address the growing burden of noncommunicable diseases (NCDs) worldwide. However, little is known about community as a concept among people living in socioeconomically disadvantaged settings and stakeholders’ interactions and engagement in NCDs prevention and management.

**Objective**: The aim of this study was to understand; (1) the meaning of community among people living in socioeconomically disadvantaged suburbs in Region Stockholm and (2) how communities interact and engage with stakeholders at local and regional levels for the prevention and management of type 2 diabetes (T2D).

**Methods**: This qualitative study was conducted in three municipalities in Region Stockholm with a high proportion of migrants. Multiple data collection methods were used, including observations of community activities; interviews with community members, representatives of public authorities and NGOs; and group interviews with healthcare providers. Data were analyzed using content analysis.

**Results**: Community was perceived as living in close proximity with shared beliefs, values and resources. Although they recognized its social and cultural diversity, community members focused more on the commonalities of living in their neighborhood and less on their differences in country of birth and languages spoken. Several mismatches between awareness of community needs and the available skills and resources among stakeholders for T2D prevention were identified. Stakeholders expressed awareness of T2D risk and interest in addressing it in a culturally appropriate manner.

**Conclusion**: Interaction between the communities and stakeholders was limited, as was engagement in T2D prevention and management. This highlights barriers in the collaboration between community, healthcare institutions and other stakeholders which consequently affect the implementation of preventive interventions. Innovative ways to link the community to the healthcare sector and other local government institutions are needed to build the capacity of health systems for T2D prevention in socioeconomically disadvantaged communities.

## Background

Noncommunicable diseases (NCDs), including diabetes, cardiovascular disease, cancer and chronic respiratory illness, are the leading cause of death and disability worldwide [1]. Diabetes alone is ranked the eighth leading cause of death and is estimated to affect more than 8% of the global adult population [2]. Type 2 diabetes (T2D) is the most common form of diabetes, a metabolic disorder characterized by hyperglycemia resulting from insufficient insulin production [2]. The condition is associated with socioeconomic status and disproportionately affects socioeconomically disadvantaged populations and immigrants in all countries particularly in high-income countries (HICs) [2].

In Sweden, where the present study was conducted, several waves of migration have led to an increasingly diverse population [3,4]. Persons born outside Sweden account for more than 12% of the population [4] and a large proportion of these come from the Middle East and Africa. These persons also constitute the highest proportion of people living in socioeconomically disadvantaged suburbs of the country’s capital, Stockholm [5,6]. T2D risk and prevalence is much higher in these areas [7,8] than in the region and the country as a whole (12–16% in adults 65 years and older compared to 5% and 6%, respectively) [7]. Noteworthy is that the Swedish healthcare system is built on the principle of universal access to quality healthcare on equal terms for the entire population [9]; thus, high burden of diabetes coupled with a strong social gradient highlights the need for a better understanding of the extent to which the needs of this population are being met.

effective strategies exist for preventing or delaying the onset of T2D [10–13] and involve modification of lifestyle factors including diet and physical activity, with a focus on empowering people and communities to bring about these changes [10,12,13].

In the *Global Action Plan for the prevention and management of noncommunicable diseases 2013–2020* [14] it is advocated that health systems shift towards prevention of NCDs and their underlying social determinants. A people-centered primary healthcare approach integrating community engagement [14] is suggested as a means to achieve this and attain Universal Health Coverage (UHC) [15]. Community engagement is defined by the WHO as a process of developing relationships that enable stakeholders to work together to address health-related issues and promote well-being to achieve positive health outcomes [16]. Such strategies are at the forefront of health promotion and disease prevention, particularly in low- and middle-income countries (LMICs) [14]. Utilization of community-clinical linkage models, such as community health workers, have been shown to be effective in providing healthcare services to persons with or at risk of chronic diseases in resource-limited settings [17,18]. However, community engagement in NCDs prevention has not been widely studied in socially disadvantaged areas in high-income settings. In Sweden, the few studies that have used community approaches are mainly population-based, with limited focus on socially disadvantaged areas [19,20]. Moreover, engagement of communities in most of these studies has generally involved participation of professionals at the municipality level in the planning and implementation of health promotion activities with limited consideration of the community members’ perspective [21]. One ongoing study has focused on lifestyle modification among a migrant population in a socioeconomically disadvantaged community in the South of Sweden [10] but, no findings from this study have yet been published.

Understanding the community context is crucial in tailoring preventive interventions and ensuring community participation [22,23]. However, the concept of community has no standard definition [23–25]. In one study, community was defined by the interactions, shared values and traditions among people who live in the same locality [23]. In another, the researchers argued that living in close proximity does not necessarily constitute a community, but rather that value systems and other cultural and social characteristics are more relevant to the context of community [24]. Other researchers suggest that the defining feature of a community is the common identity shared by its members [25]. Tindana et al. [24] add that even though the community is determined largely by shared traditions and values, communities are not static and may accommodate multiple and even conflicting interpretations of their own traditions and values.

Some research has been conducted on understanding diabetes care in socioeconomically disadvantaged settings in Sweden [26–29]. However, the focus has mainly been on understanding T2D patients’ illness perceptions [26,27] and patient and provider interactions [28,29] with less effort on understanding the community, the interactions between community stakeholders and their engagement in T2D prevention and management. The focus of this study was, therefore, to understand the meaning of community among people living in socioeconomically disadvantaged suburbs in Region Stockholm, and how these communities interact and engage with stakeholders at local and regional levels for the prevention and management of T2D.

## Methods

Data were collected between February 2015 and April 2016 by the four authors (FA, JA, IN, HMA) as part of the formative studies of a four-year implementation research project called ‘A people – centered approach to Self-Management And Reciprocal learning for the prevention and Management of Type 2 Diabetes’ (SMART2D) [30]. The SMART2D project aims at improving self-management for people at risk of or living with T2D [30].

### Study setting

The study was conducted in five socioeconomically disadvantaged suburbs in three municipalities with a high proportion of migrants in Region Stockholm [3]. The total population in these municipalities’ ranges from approximately 75, 000 to 100,000 and the proportion of people born outside Europe is between 30% and 40% [3].

Selection of the study areas was based on their initial inclusion in the SMART2D project following two main criteria: (1) Suburbs with a high proportion of immigrants. These areas were characterized by super diversity [31–33] resulting from a mix of nationalities from more than 100 countries, mostly from the Middle East (Syria, Turkey, Iraq, Iran) and Africa (mainly Somalia and Eritrea) [4]. The diversity also refers to the fact that some have newly arrived to Sweden and others have lived in the area for several decades and (2) Being socioeconomically disadvantaged defined by, high healthcare needs and high unemployment levels [5,6]. Additionally, Care Need Index (CNI) a measure socioeconomic deprivation used to allocate primary healthcare resources is much higher in these suburbs than in the affluent parts of the county [34]. The public primary healthcare structure is similar in these areas to other parts of Region Stockholm and services include; health promotion, disease prevention, curative care and rehabilitation. All these services are provided at the Primary Healthcare Centers (PHCs), directly managed by Region Stockholm [35,36]. The PHCs are available in all local residential catchment areas to serve the population and healthcare providers typically include general practitioners (GPs), nurses and other paramedical professionals (physiotherapists, occupational therapists, podiatrists) [37]. T2D care is delivered by teams of 2–3 health professionals including doctors and nurses specialized in diabetes care. Patients are referred to secondary clinics for lifestyle consultations and tertiary care including ophthalmologists, endocrinologists and nephrologists when needed [35,38].

### Study design

The study design was explorative and included several qualitative data collections methods: Community observations, group discussions and individual interviews. The data were collected in three parts, presented below. In addition, the research team conducted parallel meetings to triangulate the different parts of the data.

### Participant selection

Three sets of participants were involved in the study: (1) Community members, (2) representatives from local government, local NGOs and regional institutions and (3) primary healthcare providers and managers (Table 1). For the purpose of consistency, the term ‘stakeholders’ is used in the text to refer to participants from local government, NGOs and regional institutions.

**Table 1.** Characteristics of the study participants.

Stakeholder types	Number of participants		Data collection method (number)
**Community members**	**Male**	**Female**	
– Community members active in a formal network – Informal group leader	6	2	Group interviews (N = 4, 2 informal unrecorded)
Community members active in an informal group – Sports and gym manager – Local shop owners – Formal group leader – Community member active in a formal network	9	5	Individual interviews (N = 14, 6 informal unrecorded)
**Healthcare providers**	1	6	Group interviews (N = 3 recorded)
**Health managers**	1	3	Group interviews (N = 1 recorded)
**Local and regional stakeholders**			
**Local/municipality level** – Development strategist – Prevention coordinator – Social worker – Swedish language teacher – Member of the Swedish Diabetes Association – Manager at organization of diversity promotion work – Manager at transcultural center **Regional level** – Health educator (Region Stockholm)	4	4	Individual interviews (N = 8 recorded)

The first part of the study explored community members’ perceptions of the notion of community and their interaction with other stakeholders (local government, local NGOs and regional institutions). A step-wise approach was used, starting with the compilation of local community associations from internet sources, the civil offices (Medborgarkontor) and by word of mouth. Community groups which were not listed on internet were accessed using gate-keepers, identified through the research team’s engagement in the community between February and November 2015 [39]. Participants needed to fulfill the criteria of being born outside Europe and lived in Sweden for five years or longer. In total, 22 community members were recruited for the interviews using snowball sampling [40] (Table 1). These included men and women mainly from the Middle East (Turkey, Iran, Iraq and Syria) and Africa (Somalia, Ethiopia and Eritrea).

In the second part of the study we examined the perceptions of T2D prevention and care among healthcare providers. A purposive sample of doctors and nurses specialized in T2D care who had regular contact with T2D patients was selected to participate. Additionally, PHC managers also participated in order to get a better understanding of the structure and administration of T2D care in their respective health centers. In total, seven providers and four managers were interviewed (Table 1).

In the third part of the study, eight representatives from local government, local NGOs and regional institutions including the municipalities and Region Stockholm were purposively selected to share their experiences and strategies of engaging with socioeconomically disadvantaged communities in the prevention and management of NCDs (Table 1). All the participants had prior experience of establishing collaborations with community stakeholders in this part of Stockholm.

### Data collection

The first part of data collection process was ethnographic in nature [41] and involved approximately four months of fieldwork by FA. During this time, rapport was established with community members and leaders of local groups with support from a gatekeeper. Individual interviews (both recorded and unrecorded), natural group discussions [39] and observations were used to collect community members’ views about the meaning of community. The interviews were conducted by FA mainly in English or Arabic, the languages that most participants felt comfortable to use. In a few cases, an interpreter was used to translate from Swedish to English. The choice of individual versus natural group discussions was based on convenience and participants’ preference. Formal network leaders were individually interviewed as they were only difficult to approach except with assistance of a gatekeeper. Natural group discussions were used when individual interviews were not possible due to lack of time on the part of participants or when they preferred to participate within their ‘natural group’, be it a language class or an NGO activity such as women only sewing group [39]. A semi-structured interview guide was used to explore the defining factors of the community, needs for health support, interactions with other community stakeholders and lifestyle modification experiences. All data were collected in the community in locations chosen by participants. The recorded interviews lasted on average 47 min while informal conversations were commonly much shorter. The data were supplemented with field memos and notes from observations of everyday community life at private and public meeting points [39].

In the second part of data collection, healthcare providers involved in diabetes care in the PHCs within these communities were interviewed in their working teams of 2–3 participants. Three of the managers of the participating PHCs were also interviewed together. One of the managers who was not at the meeting was interviewed separately (Table 1). Natural group discussions were convenient for the providers. They expressed interest in sharing their experiences in their working teams and gave us an opportunity to listen to the discussions between the nurses and doctors. The interviews were moderated in Swedish by JA and HMA. The interview guide consisted of open-ended questions exploring three broad topics: Primary healthcare services offered to persons with or at risk of T2D; experiences of providing diabetes care for persons with diverse cultural backgrounds; and opportunities for collaboration with other stakeholders and T2D prevention strategies. From the provider interviews, only those parts of the data linked to community engagement and collaboration with other community stakeholders in diabetes prevention were analyzed for this paper. Other parts of the data have been published elsewhere [28]. The group discussions were recorded and these lasted on average 43 min.

In the final part of data collection, individual interviews were conducted with representatives from local authorities, municipalities and NGOs. The interviews were conducted by IN using an interview guide with questions on: Perceptions of T2D burden; involvement in health promotion and prevention of T2D; knowledge about and support for self-management and collaboration between local stakeholders. In total, six face-to-face interviews were conducted at the participants’ workplaces and two interviews were conducted by telephone. All the interviews were conducted in Swedish and tape recorded. These lasted on average 45 min.

### Data analysis

Analysis of the data was conducted by a multidisciplinary team comprised of a medical doctor (FA), two health systems researchers with backgrounds in nutrition (JA) and public health (IN), an intervention and implementation research expert (MD) and a medical anthropologist (HMA). The team had prior experience of working in both European and Non-European settings and provided a mix of insider-outsider perspectives.

The data from the three sets of participants were transcribed separately by the three data collectors (FA, JA and IN). The provider and other stakeholder interviews conducted in Swedish and English were transcribed verbatim while the community members’ interviews conducted in Arabic where directly translated and transcribed in English. Transcripts from the three sets of participants were transferred to Nvivo software and analyzed using qualitative content analysis [42–44].

**Figure 1.**
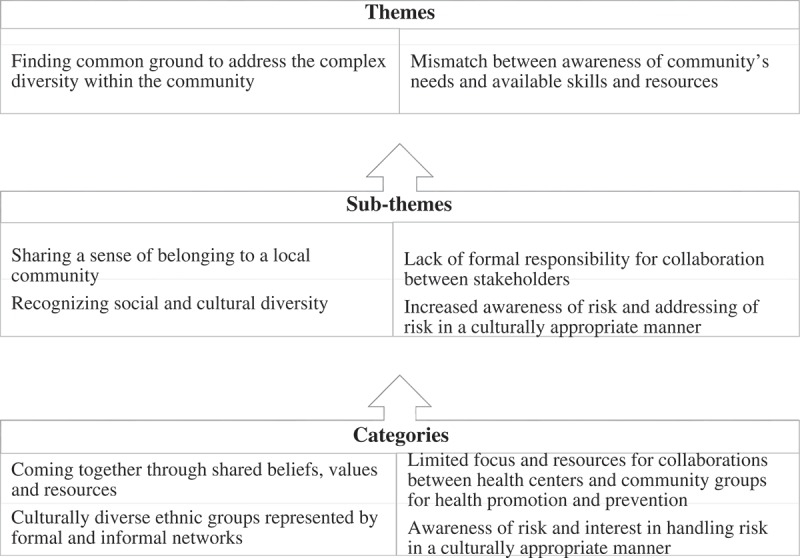
Overview of the themes, sub-themes and categories of the content analysis of the participants’ data.

Data analysis proceeded in two steps. (a) In the initial step, coding of the transcripts in their original languages was carried out to glean meaning units that described community and the interactions between stakeholders. This first step was conducted separately by FA, JA and IN supported by MD and HMA. (b) In the second step, preliminary codes were compared across the types of participants by FA, JA and IN and similar codes grouped into sub-categories and categories, which were later discussed and revised together with MD and HMA during a series of meetings. In order to enhance credibility and trustworthiness, JA examined the transcripts (peer debriefing) [39] from the interviews with the community members and the other stakeholders from local government, local NGOs and regional institutions, and together the data collection team reached a consensus on the final analysis. Triangulation of data collection methods and participant perspectives was instrumental in this final stage of analysis. A sample of the process of analysis is shown in Figure 1

## Results

Two main themes were developed: (1) Finding common ground to address the complex diversity within community and (2) Mismatch between awareness of community’s needs and available skills and resources. The first theme was informed by three sub-themes: Sharing a sense of belonging to a local community, recognizing social and cultural diversity, difficult to access the community and challenging to address a high level of diversity. The second theme is built on two sub-themes: increased awareness of the risk and addressing risk in a culturally appropriate manner and lack of formal responsibility for collaboration between stakeholders. These sub-themes and their categories are further elaborated below (Table 2).

## Finding common ground to address the complex diversity within community

This theme was built on three sub-themes: ***Sharing a sense of belonging to a local community; recognizing social and cultural diversity, difficult to access the community and challenging to address a high level of diversity***. This theme summarizes the different views the participants expressed in regard to their understanding of the meaning of community.

### Sharing a sense of belonging to a local community

Community members referred to the specific names of the areas they lived in with geographic demarcations and talked about their local community as distinct from the rest of Region Stockholm. However, there was no clear definition of the boundaries of the communities when talking about everyday social activities.

Interviews with community members revealed that despite diversity in culture, nationality, religion and languages, the overriding sentiment that ‘community‘ carried with it was of living in close proximity and caring for one another. Living in the same neighborhood was said to facilitate a common understanding between community members, bringing a sense of cohesion and belonging. In this way, community members were responsible for each other’s needs and supportive of one another.

*‘People here live very collectively. We share a lot of stuff. Neighbors knock on each other’s door. Like [borrowing] the SL card (travel card), everything is sharing and caring mentality. And you don’t care, you just knock and you’re like “Can I borrow…”’* (Female community member in formal network)

Having a common understanding among community members was considered to be far more important than having the same nationality and religion. At times it was difficult to verbalize something that came so innately.

*‘Not necessarily Iraqis, just people who are like us. So, it’s our background (living in socioeconomically disadvantaged suburbs). It’s not religion or country, but culture maybe. It’s very different here so it’s nice to have people who just understand, you know?’* (Male community member in informal network)

### Recognizing social and cultural diversity

The communities were described as dynamic and containing many formal and informal social networks of which the individual community member would only be active in a few. The networks often brought together members of the same gender, language and similar ethnic backgrounds. Informal small groups held their meetings in public places such as cafes or shopping areas. Such groups were almost exclusively male, with women mainly meeting in private homes or women’s centers. Meeting in public places including cafes was described as much cheaper than renting a private space.

The formally established groups included ethnic associations, language classes, sports clubs, women-only groups organized by local NGOs and religious groups. While there are a number of groups that are organized around the country of origin of members, such as the Somali and Kurdish organizations, the women’s centers, e.g. the sewing group, catering group and language class are driven by activities and interests. Members in the latter tend to represent multiple languages, cultures, religions and countries of origin. This extended to the origin of art displayed on the walls, music listened to and food that was eaten. None of the groups had any formal restrictions on who could join.

### Difficult to access the community

Many community members expressed a low level of trust towards outsiders from or affiliated with public authorities. They noted that they are well aware of living in socioeconomically disadvantaged areas and that they are also seen by outsiders as such.

*‘He [my dad] trusts me more than he does the authority or government. I think that is common around here. We don’t have a lot of trust in the authorities and the government, so we ask each other instead.’* (Female community member in formal group)

Access to the community groups by outsiders, i.e. people not living in these areas, including representatives of public authorities, NGOs and research institutions, was difficult without assistance from gatekeepers.

*‘Today they often say we shouldn’t speak as “we” and “them”. But we have to recognize there is a “we” and “them”. In every term. In terms of school, housing, criminality and so on.’* (Male community member active in a formal network)

Representatives from the local government acknowledged the existence of the local community groups but reported limited interaction with the members or their leaders.

*‘… I’ve heard of Kurdish association, Greek association, Somali association, there are a lot; but we don’t know them personally.’* (Female representative from the civic office at the Municipality)

Participants from local government echoed the community members’ lack of trust in authorities. In the domain of healthcare, for example, some community members were concerned about not being understood and not receiving appropriate care in the local healthcare centers. Healthcare providers, on the other hand, reported that many patients held views on treatment, food habits and physical activity that were difficult to change, and they were uncertain of how to deal with them.

To regain the community members’ trust, it was highlighted that time and resources are needed.

*‘…you have to work really hard and it is very much about… personal relationships, you have to build relationships…if someone trusts you in this area, then that person will trust others that you refer them to. You must somehow have ambassadors or key persons within the ethnic groups that are respected by the people. So, you have to work actively in the area. It does not work to come in as a stranger and believe that it will work.’* (Male representative from civic office at the Municipality).

### Challenging to address a high level of diversity

All the stakeholders recognized that these communities are culturally heterogeneous and diverse in their beliefs pertaining to lifestyle habits and health needs compared to other areas of Stockholm. It was difficult for them to identify strategies to work with groups unfamiliar to them. For example, family members’ presence during individual patients’ consultations was seen with ambivalence by healthcare providers. From a Swedish public institution perspective, it was seen as a barrier to confidential individualized care whereas from a community perspective, it was normal to have a care companion to clinic visits.

## Mismatch between awareness of community’s needs and available skills and resources

The second theme was informed by the sub-themes: ***Increased awareness of the risk, addressing risk in a culturally appropriate manner and lack of formal responsibility for collaboration between stakeholders***. The theme reflects the dilemmas and barriers the participants expressed in relation to engagement in NCDs prevention.

### Increased awareness of risk and addressing risk in a culturally appropriate manner

All participants were asked questions about the risk of NCDs, and they recognized the increasing burden of T2D and hypertension in particular. Interest was expressed in reducing the risk of T2D and suggestions were made about ways to promote health and prevent T2D, but with no clear direction and strategy on how to achieve this.

Community members reported many social problems requiring attention, T2D risk being only one of them. It was argued that strategies for addressing health behavior needed to be integrated with other social and welfare programs.

*‘.. The socio-economic difference and I mean the socio-economic class is really low here… So the unevenness in the resources can have an influence on health or lifestyle.. you have to change other things in order to be able to change lifestyle and healthcare. I don’t think you can just go and ask people to change, you have to address the root cause of their current behavior.’* (Female community member active in a formal network)

Despite these concerns, some formal networks had taken initiatives to promote health through organizing native-language seminars conducted by invited local experts. The Somali community center organized seminars once every month and T2D prevention and management had been one of the topics discussed. The Iraqi association referred to similar initiatives. Some community members were also involved in physical activity programs, such as women-only swimming groups and walking sessions, successfully started by a local NGO with financial support from the municipality.

*‘When we started this (group walks) in October last year, we found that women who said “I can’t do this, I’m not healthy”, were the ones that suggested “why don’t we walk further!”’* (Female community member active in an informal network)

Participants from the local government acknowledged the increased risk and prevalence of T2D in the communities. They expressed the need for community members to become more involved and participate in the management of their own health. It was suggested that strengthening engagement or consultation with local community networks could improve their understanding of the different needs of the respective groups pertaining to prevention and management of NCDs risk.

*‘Primarily, you have to invite the target group to define different solutions or hear, what is the need? Too often the solutions come from those who formulate policies without really knowing what the real need is.’* (Male representative from the Region Stockholm)

Culturally adapted health promotion and diabetes prevention approaches and information materials were mentioned as important. Some stakeholders expressed a need for better knowledge and understanding of the different cultural and social traditions as a way to increase utilization of offered services.

*‘I think one of the biggest problems here is that different ethnic groups are seen as one minority group…but it is not like that. Even in a country like Iraq, for example, there are several different cultural groups with different codes, traditions, and religions, so I think it is very important to know and understand the cultural differences.’* (Male from the Region Stockholm)

Community members expressed a need for better collaboration between formal institutions and the local community associations, mosques, churches and social groups. It was suggested that a more personal contact with community associations could potentially reach the most at-need population. Specific suggestions on culturally adapted health information on specific NCDs were presented, for example brochures translated to local languages.

Healthcare providers expressed a wish to support their patients to change their lifestyle through provision of health education related to healthy foods and physical activity. However, time constraints were identified as a barrier to tackling all the health concerns of community members.

### Lack of formal responsibility for collaboration between stakeholders

‘Silo’ approaches with a narrow focus and few resources available for collaboration between local government institutions were seen as barriers to improved interaction with communities. The civil offices, for example, were involved in health promotion at community level but only tackled drugs and crime prevention. There was a general perception that each sector and each institution has its own responsibility and that financial and human resources constraints were barriers to handling tasks outside their designated scope of work. The participants, however, expressed a wish and need for more structured and coordinated strategies for providing health education and other activities to the communities. They would like to approach health as one component of a group of interconnected societal challenges but are uncertain as to how to proceed without support from other sectors.

A few healthcare centers had organized outreach campaigns such as ‘Health Days’ that included health talks and group outdoor walks, but reported limited numbers of participants. Despite their efforts to support community members, healthcare providers noted the challenge of high mobility among community members. They mentioned that community members often move to better neighborhoods when they are able to or travel frequently to their home countries and are thus difficult to reach for any community targeted health activities.

## Discussion

The challenges of preventing NCDs make community engagement important to explore. This study is one of the few that describe the meaning of community and stakeholder engagement in the prevention of NCDs in socioeconomically disadvantaged settings in Sweden.

Our findings on the meaning of community resonate with a recent study conducted in a similar setting in Sweden [33] and studies from several other countries [22,23,25]. Similar to Hamed et al.’s findings on perceptions of community [33], the sentiment of community shared by the community members in the present study was of living in a socioeconomically disadvantaged neighborhood, distinct from other areas in Region Stockholm. Such sentiments lend a sense of isolation to the community members, which is further demonstrated in the consistent use of the ‘us and them’ when comparing their neighborhood to others.

Previous studies [22,23,45], have shown that having a common understanding of values among members within the community is more important than sharing the same nationality or religion. Similarly, in the present study, the community members do not see their differences in nationality or ethnicity as a barrier but rather identify with living in the same socioeconomically disadvantaged areas and sharing the same resources. Other stakeholders from the local government and public health institutions acknowledge the socioeconomic challenges in these communities but do not discuss any strategies in place to address such challenges.

Previous research has demonstrated that engaging the community in health promotion and disease prevention programs has great potential in enabling people to increase control over their health and support healthy choices [46]. However, from an implementation and health systems perspective, it is challenging to deal with diversity in multicultural settings [47]. It is particularly difficult to mobilize support and interest for health actions and to meet the needs of diverse populations while ensuring equality [47]. Intercultural approaches are suggested to be appropriate as a means to address diversity in the implementation of prevention interventions in such settings [48–50] since they accommodate population differences, but focuses on the common characteristics of the target group [48]. In practice, this would mean that interventions are based on what is common to the target population like common socioeconomic background and living in geographic proximity as in the case of the SMART2D study [30] rather than nationality/ethnicity/religion. However, such an approach does not exclude the possibility for individuals to further adapt based on their individual situation.

Although interculturalism has not been widely explored in global health research, it may offer a pragmatic solution to addressing diversity during contextualization and adaptation of health services and interventions, particularly in cities within Europe where geographically clustered complex heterogeneity is becoming a common feature today [51,52].

The difficulty of accessing community social groups and networks by other stakeholders except through gatekeepers might suggest stigmatization that might be associated with living in the socioeconomically disadvantaged neighborhood. This is expressed in the ‘us – and – them’ mentality that the community members in our study demonstrated. Other stakeholders, also reportedly worked in silos with mandates and tasks which are often restricted to specific social or health issues. This may be a contributing factor to the perceived limited interaction between the community, healthcare institutions and other public institutions, as demonstrated in our study.

Swedish studies implementing health promotion programs have mainly targeted the general population with limited focus on understanding the interactions between community stakeholders at different levels and how they engage in the health promotion activities [19–21].

Evidence suggests that community partnerships are effective in building trust and community involvement and participation in health promotion and disease prevention programs [24]. Engaging community actors in health interventions could be a potential way to improve interaction and collaboration between communities and other stakeholders and in turn extending healthcare services to the most at-need populations [17,53,54]. Community – Clinical Linkages (CCLs) is one such approach and lessons learnt from the management of other chronic diseases such as HIV indicate that integration of CCLs such as community health workers have substantially contributed to improved healthcare delivery and its cost-effectiveness [17,18], enhanced quality of life for people in poor, underserved and diverse communities [22] and strengthened human resource capacity in resource-limited settings mainly in Sub-Saharan Africa [54]. CCLs also play a key role in increasing healthcare workforce liaison with the community and enhancing community participation in chronic diseases prevention [55] in socioeconomically disadvantaged settings in HICs. Although there are limited studies in this context, these liaisons have been suggested to facilitate access to and improve the quality and cultural competence of medical care, with an emphasis on preventive and primary care [17]. CCLs could also involve outreach or community care liaisons to strengthen the link to and role of the community in the T2D prevention and management.

## Methodological strengths and weaknesses

The information power [56] of the study design and sample is assessed as relatively strong. The triangulation of multiple data collection methods including observations and informal interviews gave us access to formal and informal community practices. The credibility of the analysis was enhanced through peer-debriefing [39]. The inclusion of the perspectives of three groups of participants, the relatively long study period, collaborations between researchers with different educational backgrounds and the willingness of participants to express their views and experiences all contributed to the credibility and trustworthiness of the data. When using multiple languages, there is however a risk that some meaning in the text might have been lost in the translation of codes from Swedish to English as well as direct translation and transcription of Arabic interviews to English.

Since snowball sampling commenced with the formal and informal networks, individuals not active in these could have been missed and reaching them remains a major challenge.

Maintaining participant confidentiality meant that our description of the study context and participants’ characteristics had to be limited in detail but this did not affect the validity of our results.

The results of our study are transferable to similar settings in Sweden and other HICs in Europe characterized by highly heterogeneous populations with an increasing burden of T2D, lower socioeconomic status and a primary healthcare system that is mainly facility-based.

## Conclusion

Community to the study participants meant living in close proximity and caring for each other through shared practices. Within the community, there was a focus on the commonality of living within the specific geographical location rather than on differences of country of birth, language and ethnicity. Interaction between the community members, local government and regional institutions was limited as was their engagement in the prevention and management of T2D. The stakeholders expressed an interest in reducing T2D risk in culturally appropriate ways, but no formal responsibilities and strategies are in place to achieve this. Our findings highlight barriers in the collaboration between communities, healthcare institutions and other stakeholders at local and regional levels which should be considered when the implementing T2D prevention and management interventions. The high level of diversity within the communities in terms of culture, nationality, ethnicity, language and a plethora of formal and informal networks is a challenge to address. Further research on innovative ways to link the community to the health system and intercultural approaches to address the diversity within socioeconomically disadvantaged settings are needed.

## Paper context

T2D care in Sweden has primarily been at health facility level with minimal focus on prevention. Research on the meaning of communities, how they interact and engage with other stakeholders in prevention and management of T2D is scarce. Our findings provide a point of departure for development of community-clinical linkages to improve the capacity of the Swedish healthcare system and municipalities in dealing with the prevention and management of T2D. However, further research on innovative ways to achieve this is required.
